# Supplementary material for: Conditional chemoconnectomics (cCCTomics) as a strategy for efficient and conditional targeting of chemical transmission
Source: eLife. 2024 Apr 30;12:RP91927. doi: 10.7554/eLife.91927 (PMC11060718; doi:10.7554/eLife.91927)
Supplement: Figure 3—source data 3. [file elife-91927-fig3-data3.zip › Figure 3 source data3 part 1.zip!/Figure 3-Source data 3-part1/1175-9-CRISPResso_Report_rtnd62ko.zip!/Nucleotide_percentage_quilt_around_sgRNA_GTAGGCCACGCAGTAGACCA.pdf]

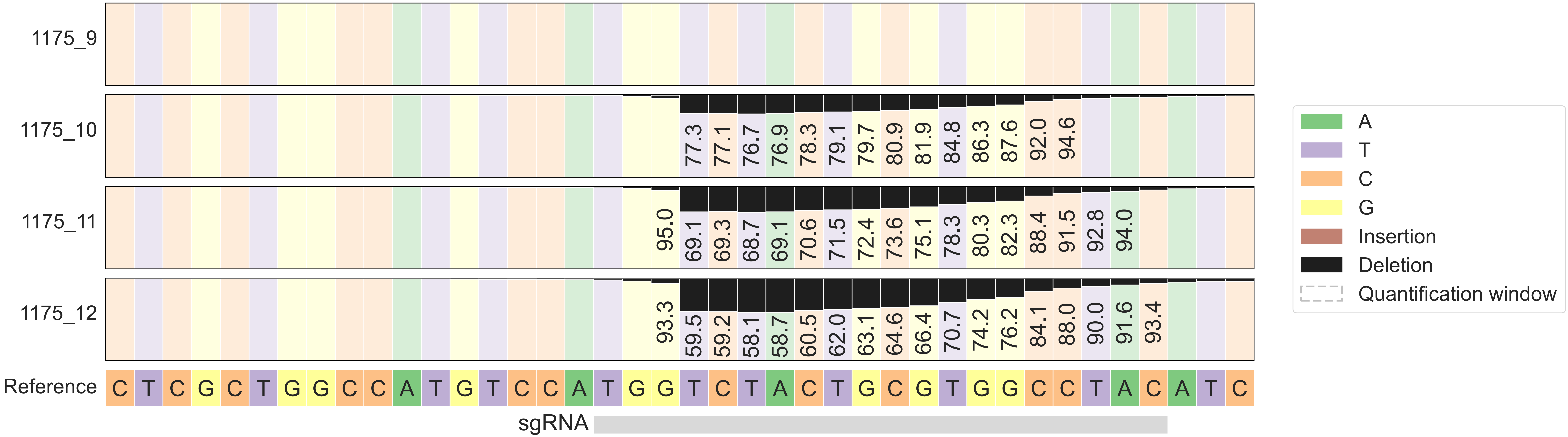
1175_9
1175_10
77.3 77.1 76.7 76.9 78.3 79.1 79.7 80.9 81.9 84.8 86.3 87.6 92.0 94.6
1175_11
95.0 69.1 69.3 68.7 69.1 70.6 71.5 72.4 73.6 75.1 78.3 80.3 82.3 88.4 91.5 92.8 94.0
1175_12
93.3 59.5 59.2 58.1 58.7 60.5 62.0 63.1 64.6 66.4 70.7 74.2 76.2 84.1 88.0 90.0 91.6 93.4
Reference
C T C G C T G G C C A T G T C C A T G G T C T A C T G C G T G G C C T A C A T C
sgRNA
A
T
C
G
Insertion
Deletion
Quantification window
